# Efficacy of probiotic in perennial allergic rhinitis under five year children: A randomized controlled trial

**DOI:** 10.12669/pjms.35.6.744

**Published:** 2019

**Authors:** Mubashir Ahmed, Abdul Gaffar Billoo, Khalid Iqbal

**Affiliations:** 1 Mubashir Ahmed, MBBS. Kharadar General Hospital, Aga Khan Road, Kharadar, Karachi, Pakistan; 2 Abdul Gaffar Billoo, FRCP. Kharadar General Hospital, Aga Khan Road, Kharadar, Karachi, Pakistan; 3 Khalid Iqbal, MBBS, DCH. Kharadar General Hospital, Aga Khan Road, Kharadar, Karachi, Pakistan

**Keywords:** Probiotic, Perennial, Allergic Rhinitis, Rhinorrhea, Cetirizine

## Abstract

**Objective:**

To determine the efficacy of probiotic (Lactobacillus Paracasei, LP-33) and compare it with cetirizine for the treatment of perennial allergic rhinitis in under five year’s children.

**Methods:**

The randomised clinical trial was conducted at Kharadar General Hospital, Karachi, from Dec 2016 to Nov 2017. Children aged 6 to 60 months, clinically presented with allergic rhinitis were included in the study. Total 212 children, randomized into intervention group A (received probiotic LP-33) and control group B (received cetirizine) for six weeks, were included in the analysis. Baseline allergic rhinitis symptoms (rhinorrhea, sneezing, nasal blocking, coughing, feeding & sleeping difficulties) were assessed after two and six weeks follow up and correlated both groups by using Pearson chi-square test. A p value of <0.05 were considered statistically significant.

**Results:**

Total 212 children were analysed, out of them 113 (53.3%) were male. Mean age of study participant was 26 ± 16.64 months and mean body weight was 10.1 ± 3.26 Kg. More than 95% cases have shown significant improvement in their baseline symptoms (rhinorrhea, sneezing, nasal blocking, coughing, feeding difficulties and sleeping difficulties) in both intervention (L-33 Probiotic) and control (Cetirizine) groups. Statistically there was no difference in effectiveness of probiotic and cetirizine treatment for perennial allergic rhinitis (P > 0.05).

**Conclusions:**

Probiotic (LP-33) was equally effective as cetirizine in under five year’s children for the treatment of perennial allergic rhinitis. Probiotic has additional benefit to treat allergic rhinitis without causing any major side effect in children reported by the study.

## INTRODUCTION

Allergic rhinitis (AR) is a common chronic inflammatory disorder of nose characterized by rhinorrhea, nasal sneezing, nasal obstruction, and itching.^1^ Increasing prevalence of allergic rhinitis have been reported worldwide.^2^ International Study of Asthma and Allergies in Childhood (ISAAC) reported, the prevalence of AR varied between 0.8 to 14.9% in 6-7 years old and 1.4 to 39.7% in 13-14 years old worldwide.^2^ In Asia, this disease affects a large population, ranging from 27% in South Korea to 32% in the United Arab Emirates^2^,^3^ Allergic rhinitis affects up to 30% of the general population.^4^ Allergic rhinitis was found to be 28.5% among children in Pakistan.^5^ Allergic rhinitis prevalence was reported 10-20% in North America, 10-15% in Europe, 20% in Thailand, 10% in Japan, and 10-26% in Indonesia.^6^

The disease process is initiated when an individual is exposed to an allergen that stimulates IgE-mediated inflammatory responses in the nasal mucosa. This leads to allergen sensitization and the development of an atopic reaction that symptomatically manifests as rhinorrhea, sneezing, nasal congestion and pruritus. Allergic rhinitis is typically a self-limited disease, medical intervention is often required for symptomatic relief. Current treatment options including allergen avoidance, antihistamines, decongestants, intranasal corticosteroids and probiotics.^7^ Use of anti-histamine for the treatment of allergic rhinitis supposed to be a primary choice but it may cause drowsiness and adversely affect cognition and performance in children.^8^ Corticosteroids are also excellent in reducing inflammation in allergic rhinitis, but in children they have a potential risk of disturbing growth and development.^6^ Probiotics, could be an alternative therapeutic modality in allergic rhinitis because of their wide availability, minimal adverse effects, and relatively low cost. Probiotics have a positive effects in the prevention and treatment of allergic disease via modifying the gut ecosystem.^9^-^11^

Probiotics are living microorganisms that confer a physiologic benefit following host administration^12^ and are found in fermented foods such as fermented beverages (Kvass), yogurt, pickles, soybean paste and dark chocolate.^13^ Lactobacillus paracassei (LP-33) is a non-steroidal dietary supplement for allergy prevention and treatment, successfully isolated by GenMont through its innovative technology platform. LP-33 has been approved to induce high level of interferon-ɣ antagonizing the action of cytokines in the allergic inflammation and resulting in the suppression of IgE secretion. Therefore, the benefits of human allergy alleviation and body’s defence enhancement can be markedly presented after oral administration of LP-33 without any site-effects.^14^

There have been limited studies on the role of probiotics in allergic rhinitis treatment, particularly from the developing countries. In Pakistan there was no data on therapeutic role of probiotic in allergic rhinitis. Current study was planned to assess the efficacy of probiotic against allergic rhinitis in under five year children and compared it with standard treatment of anti-histamine (Cetirizine).

## METHODS

Current prospective, double-blind, placebo-controlled, randomised, clinical trial was conducted at Kharadar General Hospital (KGH) during the period from December 2016 to November 2017. Children age six month to five years who clinically presented with allergic rhinitis symptoms (rhinorrhea, sneezing, nasal blocking, coughing, feeding difficulties and sleeping difficulties) were included in the trial. Informed parental consent was obtained after explaining the purpose of trial at the time of inclusion.

**Fig. 1:**
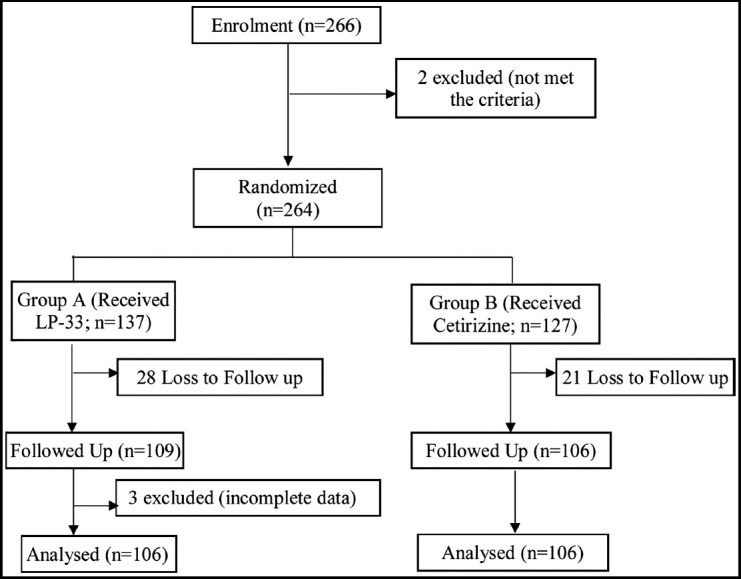
Randomization Flow chart.

Initially 264 study children were randomised into intervention group A (received LP-33) and control group B (received cetirizine) respectively. After excluding 49 lost to follow up and three incomplete data, finally 212 participants were included in the analysis (Fig. 1).

Intervention group was provided daily single dose of chewable tablet, contains two billion Colony Forming Units (2 x 109 CFU) of Lactobacillus Paracasei (LP-33), for 6 weeks. Whereas Control group received cetirizine tablet 2.5mg (< 2 years) or 5mg (2-5 years) once daily for six weeks. Tablets bottle was label with alphabetic code, provided by the manufacturer, in order to prevent the user/investigator bias. All study children parents were advised to ensure the use of provided tablets as per given instructions.

During baseline visit, study children demographic and clinical data of allergic rhinitis were taken by the attending paediatrician on pre-designed questionnaire. If children has associated co-morbidities like pneumonia, asthma, renal impairment, recent immunotherapy, hypersensitivity to study drugs or usage of antihistamines or nasal decongestants within 3-10 days were excluded from the study. Two follow up visits were made by each study children, first visit after two weeks and second visit after six weeks of baseline visit. During follow up visits paediatrician assessed the outcome of intervention in both groups and recorded the responses as full improvement (100%) or partial improvement (> 50%) or no improvement on the same questionnaire. This trial was approved by Institutional Review Board (IRB) of KGH, Karachi.

### Data Analysis

Data was entered and analyzed by using Statistical Package for Social Science (SPSS) Software version 16.0. Standard descriptive statistics were used to summarize the information recorded. Pearson’s Chi-square test was used to compare the both groups against outcome variables, improvement in allergic rhinitis symptoms, after the end of second and sixth week of intervention. A p value < 0.05 was considered statistically significant.

## RESULTS

A total of 212 study children completed the trial, 106 in each intervention Group-A and control Group-B. Of the 212 study children, 113 (53.3%) were male and 99 (46.7%) were female. Mean age of study children was 25.9 ± 16.64 months. Mean body weight was 10.1 ± 3.26 Kg.

At baseline visit, more than 90 percent of study children were presented with all cardinal symptoms of perennial allergic rhinitis. Allergic rhinitis symptoms data of each group A & B was presented in Table-I.

**Table I:** Clinical presentation of perennial allergic rhinitis at baseline visit among study participants. (N=212).

Symptoms at Baseline visit	Group A N (%)	Group B N (%)	Total N (%)
Rhinitis	106 (50.0)	106 (50.0)	212 (100.0)
Sneezing	100 (47.2)	102 (48.1)	202 (95.3)
Nasal Blocking	104 (49.0)	104 (49.0)	208 (98.0)
Coughing	105 (49.5)	105 (49.5)	210 (99.0)
Feeding difficulties	91 (42.9)	100 (47.2)	191 (90.1)
Sleeping difficulties	96 (45.3)	101 (47.6)	197 (92.9)

At first follow up visit at the end of two weeks of intervention, both group A (L-33 Probiotic) and group B (cetirizine) majority participants were shown partial improvement significantly in their baseline allergic rhinitis symptoms. Statistically there was no difference in group A (L-33 probiotic) and group B (cetirizine) treatment outcome except cough and feeding difficulties symptoms were better treated by cetirizine (P< 0.05). Table-II.

**Table II:** Clinical improvement in baseline symptoms of perennial allergic rhinitis after two weeks of intervention among study participants. (N=2012).

Symptoms after 2 weeks of intervention	Group A (Probiotics) N=106	Group B (Cetirizine) N=106	Total N=212	P-Value
Rhinitis	N=106	N=106	212	0.543
• Full improvement	14 (13.2)	9 (8.5)	23 (10.8)	
• Partial improvement	77 (72.6)	81 (76.4)	158(74.5)	
• No improvement	15 (14.2)	16 (15.1)	31 (14.6)	
Sneezing	N=100	N=102	202	0.353
• Full improvement	15 (15.0)	10 (9.8)	25 (12.4)	
• Partial improvement	75 (75.0)	84 (82.4)	158(78.2)	
• No improvement	10 (10.0)	8 (7.8)	19 (9.4)	
Nasal Blocking	N=104	N=104	208	0.611
• Full improvement	14 (13.5)	11 (10.6)	25 (12.0)	
• Partial improvement	78 (75.0)	81 (77.9)	159(76.5)	
• No improvement	12 (11.5)	12 (11.5)	24 (11.5)	
Coughing	N=105	N=105	210	0.043
• Full improvement	10 (9.5)	6 (5.7)	16 (7.6)	
• Partial improvement	78 (74.3)	85 (81.0)	163(77.6)	
• No improvement	17 (16.2)	14 (13.3)	31(14.8)	
Feeding difficulties	N=91	N=100	191	0.05
• Full improvement	11 (12.1)	9 (9.0)	20 (10.5)	
• Partial improvement	69 (75.8)	84 (84.0)	153(80.1)	
• No improvement	11 (12.1)	7 (7.0)	18 (9.4)	
Sleeping difficulties	N=96	N=101	197	0.257
• Full improvement	9 (9.4)	5 (4.9)	14 (7.1)	
• Partial improvement	75 (78.1)	85 (84.1)	160(81.2)	
• No improvement	12 (12.5)	11 (10.9)	23 (11.7)	

At second follow up visit after six weeks of intervention, both group A & B majority of participants showed full improvement in baseline allergic rhinitis symptoms.. Statistically there was no difference in treatment outcome in both groups. Probiotic and cetirizine treatment were equally effective in perennial allergic rhinitis in children under five year age (P> 0.05). See Table-III.

**Table III:** Clinical improvement in baseline symptoms of perennial allergic rhinitis after Six weeks of intervention among study participants. (N=212).

Symptoms after six weeks of intervention	Group A (Probiotic) N=106	Group B (Cetirizine) N=106	Total N=212	P-Value
Rhinitis	N=106	N=106	212	0.530
• Full improvement	86 (81.1)	92 (86.8)	178 (84.0)	
• Partial improvement	16 (15.1)	11 (10.4)	27 (12.7)	
• No improvement	4 (3.8)	3 (2.8)	7 (3.3)	
Sneezing	N=100	N=102	202	0.438
• Full improvement	84 (84.0)	91 (89.2)	175 (86.6)	
• Partial improvement	14 (14.0)	9 (8.8)	23 (11.4)	
• No improvement	2 (2.0)	2 (2.0)	4 (2.0)	
Nasal Blocking	N=104	N=104	208	0.977
• Full improvement	88 (84.6)	89 (85.5)	177 (85.1)	
• Partial improvement	13 (12.5)	12 (11.5)	25 (12.0)	
• No improvement	3 (2.9)	3 (2.9)	6 (2.9)	
Coughing	N=105	N=105	210	0.524
• Full improvement	87 (82.8)	88 (83.8)	175 (83.3)	
• Partial improvement	15 (14.3)	12 (11.4)	27 (12.9)	
• No improvement	3 (2.9)	5 (4.8)	8 (3.8)	
Feeding difficulties	N=91	N=100	191	0.473
• Full improvement	82 (90.1)	88 (88.0)	170 (89.0)	
• Partial improvement	7 (7.7)	7 (7.0)	14 (7.3)	
• No improvement	2 (2.2)	5 (5.0)	7 (3.7)	
Sleeping difficulties	N=96	N=101	197	0.757
• Full improvement	90 (93.8)	92 (91.0)	182 (92.4)	

## DISCUSSION

The main finding of current trial was “probiotic was equally effective as anti-histamine, cetirizine, for the treatment of perennial allergic rhinitis in children under five years. Current trial reported that six weeks treatment of both probiotic (LP-33) and cetirizine were shown significant improvement (95%) in symptoms of allergic rhinitis. Similar findings were also reported by a study^15^ conducted on 425 subjects with persistent allergic rhinitis showed that probiotics (LP-33) significantly improves the quality of life of study subjects with persistent allergic rhinitis.^15^ A previously published review article by Vliagofti H et al.^4^, evaluated 12 RCTs, of them 9 studies have evaluated clinical outcomes of probiotics in allergic rhinitis and showed an improvement by reporting reduced symptom severity and decreased relief medication use in allergic rhinitis patients treated with probiotics as compared to placebo. A systematic review and meta-analysis article by Zuccotti et al.^16^, documented the use of probiotic for the prevention of allergic conditions in early life, authors analysed 29 RCTs on a total of 4755 children and found a significant reduction (odds ratio 0.78, CI 0.69–0.89) in the development of allergic disease when dietary probiotic supplementation was used during pregnancy and early infancy.^16^ A recent review article on the role of probiotic supplementation in patients with allergic rhinitis by Vilà-Nadal et al.^17^, concluded that probiotics have beneficial therapeutic effects in allergic response as improvements in symptom scores and quality of life. In another systematic review on the effect of probiotics in allergic rhinitis by Zajac et al.^7^, evaluated 23 RCTs on a total 1919 patients and found a positive significant effect of probiotics on rhinitis-related quality of life (RQLQ Global Score and RQLQ Nose Score), whereas a positive but nonsignificant effect was found regarding symptom scores. Despite the lack of evidence for an improvement of symptom scores and the significant heterogeneity of these RCTs, probiotics may have beneficial effects in allergic rhinitis patients. Further studies are needed to adequately address this question.

Current trial did not report any severe or life threatening adverse effects of probiotic used in allergic rhinitis except nausea in some study children. Previous studies were also reported no severe side effect with the use of probiotic for the treatment of allergic rhinitis in children.^10^,^14^,^18^

Probiotic treatment has shown clinical improvement in allergic rhinitis symptoms and so in quality of life. The mechanism of action by which probiotics may modulate the diseases has yet to be completely defined. In mouse models, probiotics have the potential to promote T helper type 1 (Th1) immunity while suppressing Th2 responses.^19^ Other evidence suggests that probiotics may increase the predominance of regulatory T cells by altering the composition of the gut microflora.^20^ More information about the role of probiotics in the human immune response are needed to clarify through additional translational studies in the future.

### Limitations of the study

Previous RCTs conducted on efficacy of probiotics for the treatment of allergic rhinitis also performed the skin prick test to assess the house dust mite allergy and measures the serum immunoglobulin (Ig) A and E to established the allergy before intervention treatment.^21^-^25^ Current trial neither did skin test nor serum Ig A and E level because previous RCTs^18^,^21^-^25^ have reported that outcome of skin test and immunoglobulin assessment were not statistically different between probiotic and placebo groups. Other limitation includes, relatively small sample size study due to time and financial constrain, study did not utilized standard Rhinitis Quality of Life Questionnaire (RQLQ), instead used simple allergic rhinitis questionnaire, based on nasal and throat symptoms, at the time of baseline and follow up visits. Used allergic rhinitis questionnaire did not include eye and other generalized symptoms related to perennial allergic rhinitis.

## CONCLUSION

Current study provided the evidence that both probiotic and cetirizine has equally effective for the treatment of perennial allergic rhinitis in children under five years of age. Probiotic has additional benefit to treat allergic rhinitis without causing any significant side effect in children. Additional randomized controlled trials using specific probiotic strains are needed to confirm the efficacy of probiotics and to allow evidence-based recommendations.

## Authors’ Contribution:

**MA:** Designed the study, analyzed the data and prepared the manuscript.

**AGB:** Conceived, designed and final approval of the manuscript to be published.

**KI:** Responsible for collecting data, clinical assessment and revised the manuscript.
